# Opioid detection and quantification in plasma and oral fluid by LC–MS/MS

**DOI:** 10.1007/s00216-026-06336-1

**Published:** 2026-01-28

**Authors:** Luana M. Rosendo, Suzel Costa, Susana Simões, João M. Franco, Noelia Serrano Gadea, Mónica Escorial, Francisco Javier Toboso Ortega, Segundo Jiménez-García, Ana M. Peiró, Isabel Duque, Tiago Rosado, Mário Barroso, Eugenia Gallardo

**Affiliations:** 1 RISE-Health, Departamento de Ciências Médicas, Faculdade de Ciências da Saúde Universidade da Beira Interior (FCS-UBI), Av. Infante D. Henrique, 6200-506 Covilhã, Portugal; 2 Laboratório de Fármaco-Toxicologia, UBIMedical, Universidade da Beira Interior, Covilhã, Portugal; 3 Serviço de Química e Toxicologia Forenses, Instituto Nacional de Medicina Legal e Ciências Forenses-Delegação Do Sul, Lisboa, Portugal; 4 Pharmacogenetics Unit, Clinical Pharmacology Department, Alicante Institute for Health and Biomedical Research (ISABIAL), Alicante, Spain; 5 Primary Health Care C.S. Callosa d’En Sarriá, Hospital Marina Baixa, La Vila Joiosa, Spain; 6 Department of Clinical Nursing, Foundation for the Promotion of Health and Biomedical Research of the Valencian Community (Fisabio), Valencia, Spain; 7 Institute of Bioengineering, Miguel Hernández University, Elche, Spain; 8 Unidade de Dor Crónica e Medicina Paliativa, Unidade Local de Saúde de Castelo Branco, Castelo Branco, Portugal; 9 Centro Académico Clínico das Beiras (CACB)-Grupo de Problemas Relacionados com Toxicofilias, Covilhã, Portugal; 10 AlphaBiolabs, 14 Webster Court, Carina Park, Warrington, WA5 8WD UK

**Keywords:** Opioids, Plasma, Oral fluid, LC–MS/MS

## Abstract

The opioid crisis remains a significant public health concern, necessitating the development of sensitive and reliable analytical methods for drug detection. This study aimed to develop and validate a liquid chromatography–tandem mass spectrometry (LC–MS/MS) method for the simultaneous detection and quantification of fentanyl, buprenorphine, oxycodone, morphine, tramadol, and tapentadol in plasma and oral fluid. The method was validated according to FDA guidelines, assessing selectivity, linearity, precision, accuracy, matrix effect, extraction efficiency, stability, carryover, and dilution integrity. The lower limits of quantification (LLOQs) were established at 0.1 ng/mL for fentanyl, 1.2 ng/mL for tramadol, and 0.6 ng/mL for the remaining opioids, demonstrating high sensitivity. The method exhibited excellent precision and accuracy, with coefficients of variation below 15% for intra-day, inter-day, and intermediate precision analyses. Extraction efficiencies exceeded 90% for most analytes, and matrix effects remained within acceptable limits. Real-world application to authentic plasma and oral fluid samples confirmed the method’s robustness and reliability. Oral fluid concentrations were detectable across all target opioids, although plasma–oral fluid ratios showed some compound-dependent variability. These findings highlight the potential of oral fluid as a non-invasive complementary matrix to plasma for opioid monitoring, with relevant implications for forensic toxicology and clinical drug monitoring.

**Graphical Abstract:**

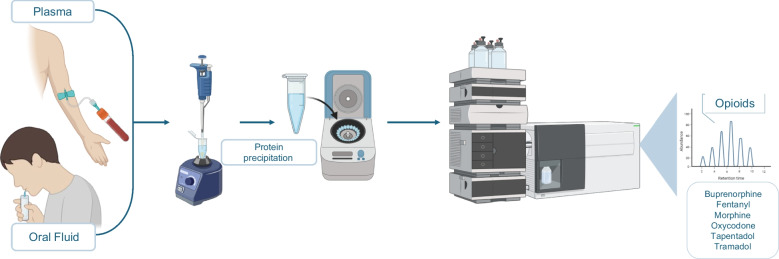

## Introduction

Opioid use continues to pose major public health and regulatory challenges worldwide, although the epidemiological context varies considerably across regions. In 2019, approximately 600,000 deaths worldwide were attributed to drug use, with close to 80% of these deaths related to opioids [1]. Notably, about 25% of these opioid-related deaths were due to overdose [2, 3]. By 2017, an estimated 40.5 million individuals globally were dependent on opioids, underscoring the widespread nature of this epidemic [4].

The proliferation of potent synthetic opioids, such as fentanyl and nitazenes, has exacerbated the crisis [3]. These substances are significantly more potent than heroin and have been linked to a surge in overdose deaths. For instance, in the USA, synthetic opioids have led to 75,000 deaths in a single year (2017) [1, 4, 5]. Similarly, the UK reported nearly 300 nitazenes-related fatalities between June 2023 and August 2024 [6, 7].

In Europe, the context differs substantially, as opioid-related challenges are characterised predominantly by the widespread therapeutic use of prescription opioids for acute and chronic pain management [8, 9]. Nonetheless, diversion, misuse, non-medical consumption, and intoxications involving these medications remain growing concerns, highlighting the need for reliable analytical tools in both clinical and forensic settings [6–9].

This alarming trend underscores the critical need for sensitive and specific analytical methods to detect and quantify opioids in various biological matrices [10–13]. Accurate analysis is essential for effective clinical monitoring, guiding treatment strategies, and informing public health interventions aimed at mitigating the impact of the opioid epidemic.

Plasma and oral fluid are two biological matrices commonly utilised for drug detection. Plasma provides reliable quantitative data on drug concentrations, serving as a gold standard for pharmacokinetic and toxicological studies. However, collecting plasma samples is invasive, requires trained personnel, and may not always be feasible in outpatient or resource-limited settings [14].

Oral fluid has emerged as a promising alternative matrix for drug testing, offering several advantages, such as ease of collection, non-invasiveness, and reduced risk of tampering. Oral fluid analysis can reflect recent drug use and may serve as a surrogate for plasma levels in certain contexts. Despite these benefits, challenges such as variability in sample collection, lower analyte concentrations, and potential matrix effects warrant a thorough comparison of its efficacy relative to plasma [15, 16].

The pharmacokinetics of opioids, including absorption, distribution, metabolism, and excretion, play a critical role in determining their detectability in biological matrices. Plasma provides a direct measure of circulating drug levels, which correlates with pharmacodynamic effects and therapeutic outcomes. Conversely, oral fluid reflects the free drug fraction and may provide a closer approximation of pharmacologically active concentrations. Variations in pH, salivary flow rate, and drug physicochemical properties can influence oral fluid drug levels, necessitating advanced analytical techniques and standardised protocols for accurate quantification [17].

Liquid chromatography-tandem mass spectrometry (LC–MS/MS) is a powerful analytical technique for opioid detection due to its high sensitivity, specificity, and ability to simultaneously quantify multiple compounds in complex biological samples. It allows for precise identification and measurement of opioids and their metabolites, ensuring accurate and reproducible results [18].

Several analytical approaches have been reported for the determination of opioids in biological matrices, most of them based on LC–MS/MS combined with extraction techniques such as solid-phase or liquid–liquid extraction. Nevertheless, the majority of these studies address only a limited range of analytes and are validated in a single biological fluid, typically plasma or urine. To our knowledge, no previously published work has described a method capable of simultaneously quantifying the six opioids investigated in this study, namely morphine, buprenorphine, tramadol, fentanyl, oxycodone, and tapentadol, in both plasma and oral fluid. This dual-matrix validation holds particular significance in the fields of forensic and clinical toxicology, where these matrices are often used complementarily. Thus, the present work was designed to develop and validate a rapid, reliable LC–MS/MS method for the simultaneous determination of six opioids in plasma and oral fluid, employing a simple, reproducible protein precipitation step suitable for implementation in routine analytical laboratories.

## Methods and materials

### Standards and reagents

The analytical standards for buprenorphine (BUP), fentanyl (FENT), morphine (MOR), oxycodone (OXY), tapentadol (TAP), tramadol (TRA), and the internal standards (ISs), methadone-d3 and morphine-d3, were purchased from Sigma-Aldrich (Steinheim, Germany). All LC–MS grade (≥ 99.0%) methanol, acetonitrile, and water were acquired from Honeywell Riedel-de-Hanën™ (Seelze, Germany). Formic acid (98–100%) was supplied by Merck (Darmstadt, Germany), and ammonium formate 1 M (p.a.) was purchased from Fluka/Sigma-Aldrich (Steinheim, Germany).

Stock solutions of each analyte were prepared at a concentration of 10 µg/mL by appropriate dilution with methanol. Working solutions of the analytes were further prepared by diluting the stock solutions with methanol: water (1:1, v/v) to achieve a final concentration of 300 ng/mL. From this intermediate solution, a series of successive dilutions were performed using methanol: water (1:1, v/v) to generate the full calibration range (0.1–300 ng/mL). A working IS solution at 500 ng/mL was also prepared using methanol: water (1:1, v/v). All solutions were stored at -20 ºC, protected from light.

Quality control (QC) samples were prepared at four concentration levels (2.3, 18.8, 75, and 300 ng/mL) within the calibration range. All QC samples were prepared from independent working solutions, separate from those used to prepare the calibration standards, to avoid analytical bias and ensure an unbiased evaluation of method performance.

### Biological samples

Blank plasma samples were obtained from a certified blood bank, the Portuguese Institute of Blood and Transplantation (Lisbon, Portugal). Oral fluid samples were collected from laboratory staff who were verified to be free from medications that could interfere with the analysis. Oral fluid samples were collected by the spitting method directly into an empty polypropylene tube, without the use of commercial collection devices or stabilising buffers. Authentic specimens were obtained from patients at centres Hospital de Ourense, and Primary Health Care C.S. Callosa d’En Sarriá through collaborative programmes with pain management departments in hospitals across Spain. The study was conducted in accordance with the Declaration of Helsinki. All participants voluntarily agreed to take part in the study after reading and signing an informed consent form. This research is part of the ethically approved project *“Opioid Monitoring and Assessment of CYP2D6, OPRM1, and COMT Phenotypes in Pain Management: Towards a Personalized Therapy”*, which received approval from the Ethics Committees of ULSCB, EPE and ISABIAL.

To ensure sample integrity and stability, the specimens were stored at -20 ºC until analysis.

### UPLC–MS/MS analysis

The UPLC–MS/MS equipment used was a Waters Acquity UPLC separation module (Waters, Milford, MA, USA), which had an integrated column oven. The Waters Acquity UPLC® HSS T3 column (100 × 2.1 mm, 1.8 μm) was maintained at 45 °C. A solvent gradient was used to produce chromatographic separation. Mobile Phase A contained LC–MS grade water, 2 mM ammonium formate, and 0.1% formic acid, while Mobile Phase B contained LC–MS grade methanol, 2 mM ammonium formate, and 0.1% formic acid. The flow rate was set to 0.4 mL/min. The chromatographic conditions for the analytes studied are summarised in Table 1.

For the first 30 s, the gradient were 90% A and 10% B. Over the next 8 min, it increased linearly to 95% B, which was maintained for three more minutes. The gradient was brought back to its starting settings at 11 min and maintained there for an additional 3 min, for a total runtime of 14 min.

The UPLC system was connected to a Waters TQ Detector (triple quadrupole mass spectrometer) fitted with an electrospray ionisation (ESI) probe. The system functioned in positive ionisation mode (ESI +), and data was obtained via multiple reaction monitoring (MRM). The following source settings were optimised: capillary voltage of 0.5 kV, extractor voltage of 2.0 V, RF lens of 0.0 V, source block temperature of 120 °C, desolvation gas (nitrogen) heated to 450 °C and delivered at 600 L/h, no cone gas and collision gas (argon) flow rate of 0.14 mL/min.

The LC–MS/MS settings were initially derived from previously optimised conditions validated for other analytes in whole blood. These parameters were subsequently adjusted and revalidated to enable the reliable detection of the selected opioids in plasma and oral fluid, ensuring methodological consistency across both matrices. This approach also guarantees compatibility and practicality within standard forensic laboratory workflows, facilitating routine implementation.

MassLynx™ V4.1 SCN 919 software was used for data collecting and system control, while TargetLynx V4.1 SCN 919 handled data processing (Waters).

**Table 1:** Chromatographic conditions of the analytes in the study

Analyte	Transitions	Cone energy	Collision energy	Rt (min)
Buprenorphine	468 → **396**/414	55	40	5.50
Fentanyl	337 → **105**/188	40	40/22	4.95
Oxycodone	316 → **298**/241	45	20/24	2.53
Morphine	286 → **165**/201	45	36/24	1.33
Tramadol	264 → **58**/ 264.2	23	14/5	3.95
Tapentadol	222 → **107**/121	40	22	4.12
Morphine-d3	289 → 201	45	24	1.31
Methadone-d3	313 → 268	27	16	6.06

Quantification transitions are indicated in bold

The 286 → 165 transition was chosen as the quantifier ion for morphine due to its superior signal intensity and enhanced signal-to-noise ratio relative to other potential fragments. The internal standard, morphine-d_3_, was monitored through the 289 → 201 transition. Despite the use of distinct product ions for the analyte and its internal standard, no matrix interferences were detected in blank plasma or oral fluid samples. Consequently, the internal standard correction remained valid, supporting accurate and reproducible quantification.

### Sample preparation

Frozen plasma samples were thawed at room temperature, as were oral fluid samples. After thawing at room temperature, the oral fluid samples were centrifuged for 5 min at 3674 × *g* before analysis.

A 0.1 mL aliquot of each sample was diluted with 0.1 mL of a MeOH: H_2_O solution (1:1, v/v), followed by the addition of 25 µL of an internal standard (IS) working solution at 500 ng/mL. Subsequently, 900 µL of ice-cold acetonitrile was added while vortexing. The samples were then agitated on a plate shaker for 10 min at 2833 × *g* and centrifuged at 5 °C for 10 min at 20,962 × *g*.

The resulting supernatant was collected in full and completely evaporated to dryness under a gentle stream of nitrogen. The dried extracts were reconstituted with 50 µL of the initial mobile phase conditions, and a 2 µL aliquot of the reconstituted solution was injected into the UPLC-MS/MS system for analysis.

The protein precipitation (PP) procedure applied in this study was developed, optimised, and validated in-house at the National Institute of Legal Medicine and Forensic Sciences (INMLCF), where it is routinely used within accredited forensic toxicology workflows for analysing various psychoactive substances in whole blood (unpublished data). Its simplicity and effectiveness made it a suitable candidate for revalidation using plasma and oral fluid samples specifically for the target opioids. This approach ensured analytical reliability while maintaining consistency with established forensic laboratory routines.

### Method validation

All validation procedures described below were conducted independently in both plasma and oral fluid matrices, using spiked blank samples prepared according to U.S. Food and Drug Administration (FDA) bioanalytical method validation guidelines [19]. The parameters evaluated included selectivity; linearity and limits (limit of detection and lower limit of quantification); intra-day, inter-day, and intermediate precision and accuracy; matrix effect; extraction efficiency; stability; carryover; and dilution integrity.

The selectivity of the method was evaluated to assess its ability to detect the target analytes in the presence of potential interferences from biological sample components. Drugs and endogenous substances that produce ions of similar high molecular weight could interfere with the detection if their retention times are comparable or if inappropriate ions are selected for monitoring. To ensure method selectivity, ten distinct blank plasma samples from a blood bank and oral fluid samples from laboratory staff members who had not consumed opioids were analysed. Additionally, two transitions for each analyte were carefully selected to avoid matrix or endogenous interferences, which were not observed in the blank samples. Spiked samples (plasma and oral fluid) were prepared and analysed using the protein precipitation (PP) extraction method. The linearity of the method was assessed using spiked samples (*n* = 5) within the concentration range of 0.1–300 ng/mL. Calibration curves were constructed by plotting the peak area ratio of each analyte to the internal standard (IS) against the analyte concentration. The acceptance criteria required a determination coefficient (*R*^2^) of at least 0.99, accuracy within ± 15% (excluding the LLOQ), and coefficients of variation (CVs) equal to or less than 15% (excluding the LLOQ).

The lower limit of quantification (LLOQ) was defined as the lowest concentration that could be measured with acceptable accuracy and precision, with a mean relative error (RE (bias)) within ± 20% of the nominal concentration and a CV less than 20%. The limit of detection (LOD) was determined by analysing five replicates of spiked samples and was defined as the lowest concentration that produced a distinct peak, clearly discernible from the blank, with a signal-to-noise ratio of at least 3.

Five replicates of blank plasma and oral fluid samples spiked with the target analytes at a minimum of four different concentration levels were examined on the same day to assess intra-day precision and accuracy. Within 5 days, inter-day precision and accuracy were assessed at a minimum of six concentrations. Intermediate precision and accuracy were assessed using four QC levels (2.3, 18.8, 75 and 300 ng/mL), analysed over five consecutive days (*n* = 3 each). Different QC levels were used for different validation parameters to comply with FDA recommendations and to ensure full coverage of the analytical range. Intra- and inter-day precision and accuracy were evaluated at multiple concentrations spanning the calibration interval, whereas intermediate precision followed a fixed set of QC levels analysed over several days to assess long-term variability. For the analysis of extraction efficiency, two sets of samples (*n* = 3) were prepared at three concentration levels (2.3, 75, and 300 ng/mL). The first set consisted of pre-extraction spiked samples, while the second set contained post-extraction spiked samples (representing 100% efficiency). Extraction efficiency was determined by calculating the ratio of the peak areas from set 1 to those of set 2.

For the matrix effect assessment, two sets of samples (*n* = 3) were prepared at the same concentrations used for the extraction efficiency analysis. Set 1 contained pure standard solutions and set 2 consisted of post-extraction spiked samples. The matrix effect was calculated as the ratio of the peak areas from set 2 to set 1.

The stability of the target analytes under various conditions was evaluated using three concentration levels corresponding to the QC concentrations (2.3, 75, and 300 ng/mL) (*n* = 3). Stability studies included processed sample stability and freeze/thaw stability. To assess processed sample stability, previously analysed extracts were reanalysed after being stored for 24 h at room temperature in the autosampler. Their concentrations were measured against the original calibration curve.

Freeze/thaw stability was evaluated by spiking plasma and oral fluid samples and storing them at − 20 ºC for 24 h. The frozen samples were then thawed at room temperature without assistance and refrozen for another 24 h under the same conditions. After three freeze/thaw cycles, the samples were extracted, analysed, and compared to freshly prepared and tested samples. The analytes were considered stable if the CV between the two sets of samples was below 15%.

Carryover was assessed following the extraction of a sample spiked at the upper limit of quantitation (ULOQ), 300 ng/mL, which represents the highest analyte concentration that can be quantified with acceptable accuracy and precision. Immediately after this injection, the initial mobile phase composition was injected under the same chromatographic conditions. Carryover was considered absent when no peaks were observed at the retention times and selected ions corresponding to the target analytes. This approach complies with FDA recommendations, which state that carryover should not exceed 20% of the LLOQ.

The dilution test was conducted by preparing high-concentration samples in matrix and diluting them to make analyte concentrations fall within the calibration range. A stock solution of 2000 ng/mL was diluted tenfold to obtain a final concentration of 200 ng/mL, while a secondary solution of 500 ng/mL was diluted fivefold and twofold to achieve final concentrations of 100 ng/mL and 250 ng/mL, respectively. All dilutions were performed using Milli-Q water. Each diluted sample was analysed in quintuplicate, and the CV% and RE% were calculated to evaluate method performance. The method was valid if CV% and RE% remained within ± 15% of the expected values.

## Results and discussion

### Selectivity

Selectivity was confirmed, as no endogenous or matrix-related interferences were observed at the retention times or MRM transitions of the target analytes in any of the blank plasma or oral fluid samples.

### Linearity and calibration model

The procedure demonstrated linearity for fentanyl in the range of 0.1 to 300 ng/mL; for buprenorphine, oxycodone, morphine, and tapentadol from 0.6 to 300 ng/mL; and for tramadol from 1.2 to 300 ng/mL. To account for heteroscedasticity, weighted least squares regressions were applied. Six weighting factors (1/√x, 1/x, 1/x^2^, 1/√y, 1/y, 1/y^2^) were tested, and the factor yielding the best fit was selected. The optimal weighting factor was chosen based on the lowest sum of errors and a mean *R*^2^ of at least 0.99 (Table 2). The selected weighted least squares regression confirmed linearity, with the RE between measured and spiked concentrations remaining within a ± 15% range across all concentrations. Precision was also satisfactory, with CVs typically below 15%.

**Table 2:** Linearity data (*n* = 5) of plasma and oral fluid (1/x weighting was used for all analytes)

	Analyte	Linear range (ng/mL)	Linearity		*R*^2^ª	LOD (ng/mL)	LLOQ (ng/mL)
			Slopeª	Interceptª			
Plasma	Buprenorphine	0.6–300	0.00025 ± 0.00002	0.00108 ± 0.00110	0.99567 ± 0.0307	0.6	0.6
	Oxycodone	0.6–300	0.00284 ± 0.00040	0.00869 ± 0.00617	0.99813 ± 0.00051	0.3	0.6
	Fentanyl	0.1–300	0.01230 ± 0.00094	0.03857 ± 0.03356	0.99746 ± 0.00157	0.1	0.1
	Morphine	0.6–300	0.00701 ± 0.00070	0.03614 ± 0.05845	0.99886 ± 0.00063	0.6	0.6
	Tapentadol	0.6–300	0.01247 ± 0.00138	0.04584 ± 0.02438	0.99618 ± 0.00119	0.3	0.6
	Tramadol	1.2–300	0.00970 ± 0.00097	0.04261 ± 0.01622	0.99660 ± 0.00175	0.6	1.2
Oral fluid	Buprenorphine	0.6–300	0.00023 ± 0.00001	−0.00001 ± 0.00005	0.99616 ± 0.00191	0.6	0.6
	Oxycodone	0.6–300	0.00254 ± 0.00014	0.00014 ± 0.00014	0.99589 ± 0.00215	0.3	0.6
	Fentanyl	0.1–300	0.01169 ± 0.00062	0.00171 ± 0.00194	0.99757 ± 0.00166	0.1	0.1
	Morphine	0.6–300	0.00728 ± 0.00043	0.00130 ± 0.00171	0.99864 ± 0.00076	0.6	0.6
	Tapentadol	0.6–300	0.01035 ± 0.00117	0.00152 ± 0.00307	0.99835 ± 0.00065	0.3	0.6
	Tramadol	1.2–300	0.00964 ± 0.00089	0.00029 ± 0.00379	0.99768 ± 0.00230	0.6	1.2

ªMean ± standard deviation

### Limits of detection and quantification

According to existing guidelines, the detection of opioids in biological matrices requires highly sensitive and specific analytical techniques. The Substance Abuse and Mental Health Services Administration (SAMHSA) [20] and the European Workplace Drug Testing Society (EWDTS) [21] provide recommended cut-off concentrations for opioid detection. SAMHSA guidelines set the screening cut-off for fentanyl in oral fluid at 1 ng/mL, with a confirmation cut-off of 0.5 ng/mL. Morphine and oxycodone have screening cut-offs of 30 ng/mL and confirmation cut-offs of 15 ng/mL [20]. EWDTS sets the confirmation cut-offs at 15 ng/mL for morphine and oxycodone, 20 ng/mL for tramadol, and 1 ng/mL for buprenorphine [21].

**Fig. 1:**
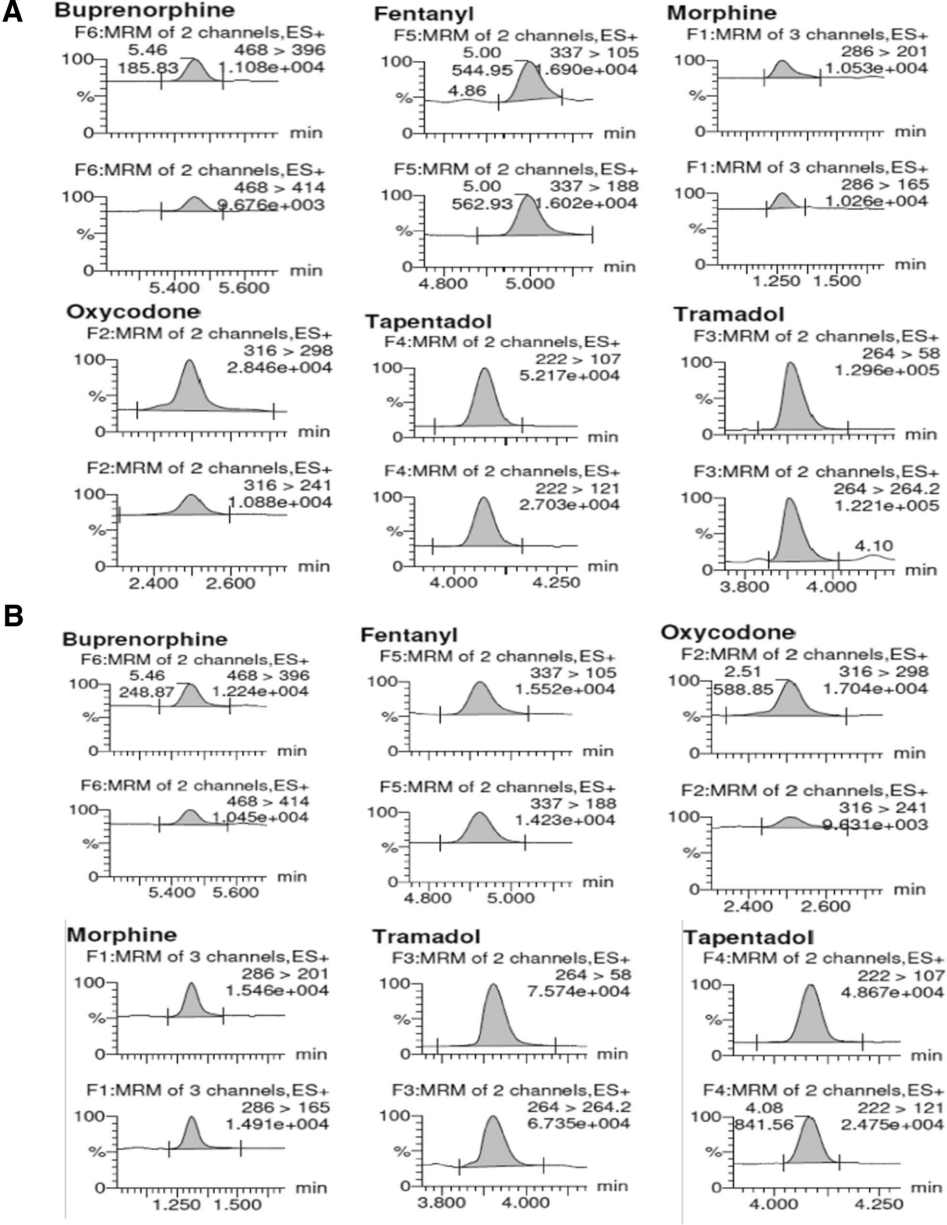
LLOQ of the opioids in plasma (**A**) and oral fluid (**B**)

The method developed in this study achieved markedly low LLOQ, 0.1 ng/mL for fentanyl, 0.6 ng/mL for buprenorphine, tapentadol, morphine and oxycodone, and 1.2 ng/mL for tramadol, across both plasma and oral fluid (Fig. 1). These values are well below the cut-offs established by SAMHSA and EWDTS, demonstrating that the method provides sufficient sensitivity for confirmatory analysis of opioids in oral fluid. The detection limits (LOD) were generally the same as the LLOQ, except for tramadol, oxycodone, and tapentadol, where they were 0.6 ng/mL and 0.3 ng/mL, respectively.

When compared with previously published LC–MS/MS methods using more complex extraction techniques, such as liquid–liquid extraction (LLE), solid-phase extraction (SPE), microextraction by packed sorbent (MEPS), and thin-film microextraction (TFME), the present method consistently demonstrates improved or comparable sensitivity. In oral fluid, LLE methods have reported LLOQs of 1 ng/mL for fentanyl and 10 ng/mL for morphine and tramadol [22], while MEPS yielded a morphine LOQ of 5 ng/mL [23]. SPE methods have also reported LOQs of 10 ng/mL for morphine [24] and 1.5 ng/mL for oxymorphone and morphine [25]. In contrast, the current method achieved substantially lower LLOQs of 0.6 ng/mL for morphine, 1.2 ng/mL for tramadol and 0.1 ng/mL for fentanyl. Similarly, for buprenorphine, previously reported LLOQs range from 1 ng/mL using LLE [22] to 0.5 ng/mL with MEPS [26].

A similar trend is observed in plasma. Although the most sensitive published LC–MS/MS method quantified fentanyl at 0.030 ng/mL [27]. Typical SPE and PP workflows report higher LLOQs, such as 0.2–1 ng/mL for fentanyl [28, 29], 1.7 ng/mL for buprenorphine [30], and 1–5.3 ng/mL for oxycodone [30, 31]. Tramadol LLOQs in plasma typically range from 1 to 10 ng/mL using SPE or PP [28, 32]. For morphine, SPE-based methods have reported LLOQs between 0.25 [33] and 4.7 ng/mL [13]. The present method outperforms most of these, achieving LLOQs of 0.6 ng/mL for buprenorphine, morphine and oxycodone, 1.2 ng/mL for tramadol, and 0.1 ng/mL for fentanyl.

To ensure a balanced assessment, the performance of the present method was also compared with LC–MS/MS methods using protein precipitation (PP), which represents the most directly comparable extraction strategy. For plasma, recent PP studies have reported LOQs of 1 ng/mL for morphine [34], while other PP methods achieved LOQs of 0.2 ng/mL for oxycodone and fentanyl and 10 ng/mL for tramadol [28]. Older, highly targeted mono-analyte PP methods reported LOQs of 0.05–0.1 ng/mL for buprenorphine [35, 36], and 0.025 ng/mL for oxycodone [37]. Compared with these values, the present method demonstrates substantially higher sensitivity for morphine, tramadol and oxycodone (8–16-fold improvement relative to the more recent PP workflows) and improved or comparable sensitivity for fentanyl and buprenorphine.

For oral fluid, PP-based LC–MS/MS methods are extremely limited. The only study identified, reported LOQs of 1 ng/mL for morphine and 0.5 ng/mL for buprenorphine [38]. The present method achieved LOQs of 0.6 ng/mL for both analytes, indicating improved sensitivity for morphine and comparable performance for buprenorphine. No PP workflows have been reported for oxycodone, fentanyl, tramadol or tapentadol in oral fluid, making this study the first approach to quantify a six-opioid panel in this matrix using a PP approach.

Overall, the findings demonstrate that the developed method provides superior sensitivity relative to most published analytical workflows, including those employing more laborious extraction techniques such as SPE, LLE or MEPS. Its combination of low sample volume, minimal sample preparation (PP), short processing time, and low LLOQs across six opioids positions this workflow as a robust option for clinical toxicology, therapeutic monitoring, and forensic screening where trace-level detection is required.

Overall, the method offers high sensitivity, robustness and broad applicability, supporting its suitability for clinical toxicology and therapeutic monitoring. Although validation was performed using clinical specimens, the analytical performance obtained is fully compatible with the sensitivity requirements typically applied in forensic toxicology, where the concentrations in drug-related deaths are usually higher. The method, therefore, has potential applicability in both clinical and forensic settings, provided that laboratory-specific validation procedures are followed.

### Intra-day, inter-day, and intermediate precision and accuracy

The method showed consistent precision and accuracy across all validation levels in both matrices. In plasma (Table 3), intra-day CV values ranged from 1.00 to 11.17%, with RE values within ± 13%. Inter-day precision remained robust, with CVs between 1.22 and 14.00% and RE within ± 10%. Intermediate precision over 5 days showed CVs between 1.66 and 11.14%, with RE within ± 6%.

Similar performance was observed in oral fluid (Table 4). Intra-day CV values ranged from 0.40 to 6.36%, with RE within ± 15%. Inter-day CVs varied between 1.22 and 13.70% and RE between –9.04 and 6.18%. Intermediate precision yielded CVs from 3.51 to 11.11% and RE between –4.80 and 6.48%.

Overall, the aligned results across intra-day, inter-day, and intermediate assessments confirm that the method is precise and accurate in both plasma and oral fluid. This performance supports its suitability for routine quantitative analysis, therapeutic monitoring, and pharmacokinetic applications.

**Table 3:** Inter-day, intra-day, and intermediate precision and accuracy of plasma samples

Analyte	Concentration (ng/mL)	Plasma					
		Intra-day (*n* = 5)		Inter-day (*n* = 5)		Intermediate (*n* = 15)	
		CV (%)	RE (%)	CV (%)	RE (%)	CV (%)	RE (%)
Buprenorphine	0.6 1.2 2.3 4.7 9.4 18.8 37.5 75 150 300	7.16 - 11.17 - - 7.33 - 6.23 - 4.03	-6.29 - 3.98 - - -8.43 - 5.53 - 7.91	14.00 - 9.08 - 10.33 12.66 - 7.09 8.57 5.00	4.43 - 1.21 - 0.00 1.59 - 0.37 0.00 0.21	- - 3.85 - - 8.16 - 7.90 - 9.23	- - -0.12 - - -0.36 - 1.56 - 3.12
Fentanyl	0.1 0.3 0.6 1.2 2.3 4.7 9.4 18.8 37.5 75 150 300	7.60 - - - 5.38 - - 4.00 - 2.00 - 2.38	3.65 - - - 12.97 - - -6.73 - 3.40 - 0.46	13.27 - 6.36 - 5.53 - 6.92 8.53 - 5.86 5.97 2.55	1.18 - 1.48 - 6.18 - 2.08 -4.29 - -2.04 -0.57 1.41	3.47 - - - 6.84 - - 8.53 - 6.11 - 4.40	1.03 - - - 2.68 - - -2.68 - 0.83 - 2.59
Morphine	0.6 1.2 2.3 4.7 9.4 18.8 37.5 75 150 300	1.00 - 9.66 - - 6.56 - 7.51 - 8.49	5.26 - -5.32 - - -10.82 - 3.16 - 2.80	13.30 - 11.62 - 7.71 7.47 - 4.01 2.77 1.22	4.20 - -9.04 - -1.83 -4.63 - -1.74 -0.41 0.94	- - 3.91 - - 9.02 - 2.83 - 4.63	- - -3.34 - - -4.13 - 0.20 - 2.77
Oxycodone	0.6 1.2 2.3 4.7 9.4 18.8 37.5 75 150 300	8.64 - 7.85 - - 4.63 - 2.69 - 3.65	1.63 - 5.71 - - -11.32 - 6.78 - 1.64	13.47 - 8.38 7.81 5.64 9.70 - 5.63 5.04 2.70	-0.98 - 1.07 -0.94 -2.69 -0.67 - -1.18 1.40 -0.39	- - 5.58 - - 11.14 - 7.53 - 5.10	- - 2.27 - - -3.00 - 1.12 - 1.83
Tapentadol	0.6 1.2 2.3 4.7 9.4 18.8 37.5 75 150 300	3.01 - 2.70 - - 5.29 - 4.86 - 3.05	-6.77 - 1.14 - - -7.36 - 8.08 - -4.67	10.42 - 9.37 - 4.67 5.89 - 11.27 4.99 4.27	3.22 - 2.73 - -6.73 -4.88 - 0.28 -0.38 1.03	- - 3.78 - - 5.60 - 8.05 - 6.59	- - 2.04 - - -5.06 - 1.40 - 0.50
Tramadol	1.2 2.3 4.7 9.4 18.8 37.5 75 150 300	10.30 9.79 - - 6.02 - 1.53 - 4.17	-4.16 -4.86 - - -7.70 - 4.07 - 1.70	5.85 - - 8.42 9.49 - 7.65 4.52 3.77	5.28 - - -1.42 -2.32 - -0.56 4.28 -1.52	- 2.88 - - 8.15 - 7.46 - 1.66	- -1.42 - - -4.91 - -1.21 - 0.73

**Table 4:** Inter-day, intra-day, and intermediate precision and accuracy of oral fluid samples

Analyte	Concentration (ng/mL)	Oral Fluid					
		Intra-day (*n* = 5)		Inter-day (*n* = 5)		Intermediate (*n* = 15)	
		CV (%)	RE (%)	CV (%)	RE (%)	CV (%)	RE (%)
Buprenorphine	0.6 1.2 2.3 4.7 9.4 18.8 37.5 75 150 300	2.68 - 2.91 - - 0.98 - 1.83 - 3.68	10.99 - -5.60 - - 4.83 - 10.25 - -9.34	13.52 - 7.48 - 7.32 11.35 - 9.72 2.88 3.10	1.55 - 3.10 - -3.21 -3.43 - 2.45 3.34 -1.81	- - 11.10 - - 10.55 - 10.95 - 7.71	- - 0.99 - - -2.07 - 5.80 - -4.61
Fentanyl	0.1 0.3 0.6 1.2 2.3 4.7 9.4 18.8 37.5 75 150 300	2.59 - - - 1.35 - - 1.97 - 3.49 - 3.37	13.06 - - - -9.67 - - 7.12 - 9.95 - -7.57	13.70 - 4.88 - 6.70 - 6.65 7.18 - 7.77 5.40 2.93	1.50 - -1.47 - 2.77 - -1.05 1.91 - 0.30 2.47 -1.61	10.64 - - - 7.55 - - 6.11 - 8.24 - 9.09	2.69 - - - 4.32 - - 4.04 - 4.83 - -3.13
Morphine	0.6 1.2 2.3 4.7 9.4 18.8 37.5 75 150 300	6.36 - 3.35 - - 4.32 - 0.76 - 3.79	14.62 - -9.15 - - 4.73 - 9.64 - -7.61	8.21 - 7.41 - 9.31 6.65 - 7.53 2.33 1.71	-8.18 - 6.49 - 0.34 -1.12 - 0.66 0.12 0.38	- - 9.89 - - 5.49 - 9.41 - 4.40	- - 2.72 - - 0.08 - 2.46 - -1.11
Oxycodone	0.6 1.2 2.3 4.7 9.4 18.8 37.5 75 150 300	1.69 - 0.73 - - 0.40 - 1.58 - 2.58	0.87 - -8.75 - - 7.31 - 10.69 - -9.50	13.19 - 7.81 - 4.67 10.60 - 6.90 5.30 4.82	-3.27 - 3.91 - -2.49 1.02 - -0.19 2.87 -1.03	- - 8.78 - - 9.36 - 8.93 - 3.51	- - 4.65 - - -0.22 - 3.51 - -4.80
Tapentadol	0.6 1.2 2.3 4.7 9.4 18.8 37.5 75 150 300	0.94 - 2.19 - - 1.31 - 1.74 - 3.01	13.22 - -8.05 - - 1.59 - 6.00 - -12.97	14.26 - 8.57 - 9.24 6.62 - 6.85 2.13 2.67	-3.60 - 1.92 - -0.46 2.12 - 1.24 0.78 -0.78	- - 7.71 - - 5.99 - 8.75 - 10.09	- - 6.48 - - 2.79 - 5.24 - -0.51
Tramadol	1.2 2.3 4.7 9.4 18.8 37.5 75 150 300	2.69 1.44 - - 4.51 - 2.74 - 1.93	9.06 -2.00 - - 1.15 - 9.40 - 7.40	12.35 8.74 - 9.69 9.94 - 8.28 5.08 2.09	4.47 4.19 - -1.80 -2.56 - -1.82 0.98 0.31	- 8.78 - - 10.03 - 10.30 - 9.35	- 4.82 - - 0.02 - 1.97 - 0.68

### Matrix effect and extraction efficiency

The extraction efficiency and matrix effect of the target analytes were assessed in pooled blank samples of plasma and oral fluid (10 different origins each) at three concentration levels (2.3 ng/mL, 75 ng/mL, and 300 ng/mL), with the results presented in Tables 5 and 6, respectively. In plasma (Table 5), most analytes exhibited recoveries exceeding 90%, with the exception of morphine, which ranged from 55.46 to 86.37%, suggesting potential analyte loss during extraction.

In oral fluid (Table 6), recoveries were generally higher, with most analytes exceeding 100%, except for morphine at the lowest concentration (81.37%). Variations in recovery may be attributed to differences in physicochemical properties, including polarity and protein binding affinity, which influence extraction efficiency. Notably, tramadol, tapentadol, and buprenorphine demonstrated consistent recoveries near or above 100% in both matrices, indicating efficient and reproducible extraction.

Matrix effects were evaluated to assess potential ion suppression or enhancement due to endogenous sample components, with results summarised in Table 5 (plasma) and Table 6 (oral fluid). In plasma, oxycodone, morphine, and tapentadol exhibited ion suppression, with matrix effect values ranging from 72.41 to 90.50%, whereas buprenorphine showed slight ion enhancement, reaching 113.73% at 75 ng/mL. In oral fluid, matrix effects were more pronounced for certain analytes, particularly morphine and oxycodone, which exhibited variability across concentrations (83.73–110.59%). Despite these fluctuations, matrix effects remained within acceptable limits, with CV% values below 15%, indicating method robustness. Differences in matrix effects between plasma and oral fluid are expected due to variations in biological composition, which may influence ion suppression or enhancement during analysis.

These findings confirm that the analytical method ensures reliable extraction efficiency and minimal matrix effects, supporting accurate quantification of the target analytes in both plasma and oral fluid. The observed variability in morphine and oxycodone highlights the necessity of thorough method validation to account for potential matrix-induced ion suppression or enhancement, ensuring the accuracy and precision of the assay.

**Table 5:** Extraction efficiency and Matrix effect (%) of the target analytes (*n* = 3) in plasma samples

Analyte	Recovery (%) ª			Matrix effect (%) ª		
	2.3 ng/mL	75 ng/mL	300 ng/mL	2.3 ng/mL	75 ng/mL	300 ng/mL
Buprenorphine	99.25 ± 5.90	97.02 ± 1.54	99.70 ± 0.40	111.87 ± 2.48	113.73 ± 1.69	104.53 ± 3.74
Oxycodone	91.77 ± 2.22	96.28 ± 3.19	107.92 ± 2.80	80.71 ± 2.35	88.45 ± 5.22	84.03 ± 5.74
Fentanyl	112.70 ± 2.00	111.68 ± 1.63	103.17 ± 2.10	98.60 ± 2.19	92.99 ± 5.46	85.53 ± 5.36
Morphine	55.46 ± 2.80	62.16 ± 1.83	86.37 ± 3.14	73.03 ± 1.42	72.41 ± 3.63	74.21 ± 2.81
Tapentadol	105.39 ± 7.13	105.71 ± 1.61	104.59 ± 4.24	88.71 ± 1.12	83.84 ± 6.58	90.50 ± 5.25
Tramadol	106.97 ± 12.09	107.40 ± 9.32	108.19 ± 6.65	106.92 ± 4.58	103.88 ± 2.20	107.72 ± 9.43

ª Mean ± standard deviation

**Table 6:** Extraction efficiency and Matrix effect (%) of the target analytes (*n* = 3) in oral fluid samples

Analyte	Recovery (%) ª			Matrix effect (%) ª		
	2.3 ng/mL	75 ng/mL	300 ng/mL	2.3 ng/mL	75 ng/mL	300 ng/mL
Buprenorphine	111.31 ± 6.14	117.28 ± 2.32	105.69 ± 4.75	105.72 ± 14.58	96.11 ± 2.89	108.40 ± 7.38
Oxycodone	97.85 ± 4.68	114.66 ± 4.39	109.12 ± 3.79	107.46 ± 6.46	83.43 ± 8.40	96.39 ± 7.25
Fentanyl	102.63 ± 9.92	106.49 ± 7.46	113.87 ± 5.42	110.66 ± 3.80	96.40 ± 5.55	95.27 ± 3.30
Morphine	81.37 ± 2.80	113.18 ± 5.08	99.74 ± 5.74	96.25 ± 1.12	110.59 ± 1.49	83.73 ± 9.65
Tapentadol	107.62 ± 5.59	118.62 ± 0.49	112.03 ± 3.85	104.23 ± 5.69	90.22 ± 11.52	111.98 ± 4.29
Tramadol	110.32 ± 6.81	116.95 ± 2.68	114.43 ± 1.65	113.24 ± 1.92	97.16 ± 10.91	114.83 ± 4.89

ª Mean ± standard deviation

### Stability

The stability of the target analytes was studied under specific conditions and time intervals that mimic those typically used for the collection and storage of biological samples. In this study, both freeze/thaw stability and stability in processed samples were evaluated to assess the behaviour of the analytes. The evaluation of processed sample stability was conducted at the same concentrations as the quality control (QC) samples (*n* = 3), where previously analysed samples were reanalysed after being stored in the autosampler for 24 h. The analyte concentrations were determined based on the original calibration curve, yielding CVs ranging from 0.30 to 14.25%, with REs falling within a range of 0.68 to 13.38% (Table 7). For freeze/thaw stability, the stability of the analytes was investigated at three concentration levels (*n* = 3). Spiked samples were frozen at -20 °C for 24 h and then thawed at room temperature without assistance. The samples were then refrozen for 24 h, completing one freeze/thaw cycle. A total of three freeze/thaw cycles were performed, after which the samples were extracted, analysed, and compared with freshly prepared samples. All compounds were considered stable after three freeze/thaw cycles, as the CVs achieved were below 13.31% and the RE remained within a 14.66% interval.

In comparing the results between plasma and oral fluid, some differences in the stability of the analytes were observed. For processed samples, plasma typically exhibited higher variability compared to oral fluid. For example, BUP at 2.3 ng/mL in plasma had a CV of 9.97% and an RE of 2.06%, while in oral fluid, the CV was 1.95% and the RE was 9.76%. Similarly, OXY at 2.3 ng/mL showed a CV of 8.52% in plasma with an RE of 7.47%, compared to oral fluid, which had a CV of 0.77% and an RE of 12.65%. On the contrary, TRA had a CV lower in plasma samples than oral fluid samples. The REs for processed samples in plasma were generally lower than those in oral fluid.

Regarding freeze/thaw stability, the plasma samples also showed higher variability compared to oral fluid for most analytes. For instance, buprenorphine at 75 ng/mL had a CV of 12.35% in plasma with an RE of 3.92%, whereas in oral fluid, the CV was 6.12% with an RE of 5.50%. Similarly, fentanyl at 2.3 ng/mL showed a CV of 1.84% and an RE of 12.90% in plasma, compared to oral fluid, which had a CV of 5.29% and an RE of 4.14%. In contrast, analytes such as oxycodone at 2.3 ng/mL displayed a similar stability profile in both plasma and oral fluid, with CVs of 5.11% and 7.55%, respectively, and REs of 0.70% in plasma and 9.87% in oral fluid.

Overall, oral fluid demonstrated more stable results with lower variability in both processed sample stability and freeze/thaw stability compared to plasma, suggesting that oral fluid may be more reliable for certain analyte measurements under these conditions.

In all evaluation stability tests, all analytes were deemed stable in both plasma and oral fluid (Table 7).

**Table 7:** Stability evaluation

Analyte	Concentration (ng/mL)	Plasma				Oral fluid			
		Processed samples (*n* = 3)		Freeze/thaw stability (*n* = 3)		Processed samples (*n* = 3)		Freeze/thaw stability (*n *= 3)	
		CV (%)	RE (%)	CV (%)	RE (%)	CV (%)	RE (%)	CV (%)	RE (%)
Buprenorphine	2.3	9.97	-2.06	10.59	4.56	1.95	9.76	13.31	1.43
	75	5.48	-8.20	12.35	-3.92	3.44	8.31	6.12	5.50
	300	6.91	-0.54	3.62	-10.06	4.53	-3.55	4.41	-3.08
Oxycodone	2.3	8.52	-7.47	5.11	0.70	0.77	12.65	7.55	9.87
	75	3.31	7.75	5.44	-1.70	3.03	5.69	0.30	14.66
	300	1.23	10.26	2.08	-9.05	14.25	-2.06	3.82	6.45
Fentanyl	2.3	4.92	5.01	1.84	-12.90	2.50	9.93	5.29	-4.14
	75	3.61	9.70	3.87	-0.97	6.36	4.61	0.40	4.82
	300	1.63	11.75	4.03	-8.58	10.72	-1.95	1.36	-9.24
Morphine	2.3	2.71	10.13	4.93	5.11	3.79	0.78	3.96	-10.41
	75	8.57	0.66	2.61	-3.48	4.99	-0.68	4.19	0.80
	300	8.45	4.85	7.26	-8.34	5.37	-4.99	0.53	11.86
Tapentadol	2.3	1.90	6.33	10.87	2.70	0.30	10.91	4.50	-0.63
	75	1.57	6.15	6.44	-8.65	2.96	5.63	1.10	10.00
	300	0.51	12.67	0.60	-11.73	9.22	-2.71	3.21	1.42
Tramadol	2.3	2.32	-3.98	9.09	4.42	3.63	5.29	0.54	-4.01
	75	0.35	11.80	10.01	-7.43	7.23	-6.02	5.24	-1.26
	300	1.56	13.38	1.91	-14.02	8.67	-6.82	3.98	-4.83

### Carryover

The potential for carryover was assessed by injecting the initial mobile phase composition, following the injection of the ULOQ sample using UPLC-MS/MS. No signals were detected at the retention time or corresponding selection ions for the target analytes in the methanol injections. These results indicate that no carryover effect was observed, confirming the absence of contamination or interference from previously analysed samples.

### Dilution test

The adequacy of sample dilution for highly concentrated samples was tested by diluting these samples with a blank matrix and then multiplying the obtained concentrations by the dilution factor. The results for plasma and oral fluid are presented in Table 8, showing the coefficient of variation (CV%) and relative error (RE%) for each dilution.

In plasma samples, the CV% values remained relatively low across all analytes and dilution factors, indicating good precision. For example, buprenorphine and fentanyl exhibited CV% values ranging from 2.81 to 8.21% at 100 ng/mL, suggesting minimal variation in the measured concentrations. However, some analytes, such as morphine, demonstrated higher CV% at certain concentration levels, such as 7.45% and 8.90% at 250 ng/mL and 200 ng/mL, respectively. The RE% in plasma was also well within the typical range for analytical methods, with deviations between -11.22 and 11.22%, further reinforcing the accuracy of the dilutions.

In oral fluid samples, the CV% values were generally higher than in plasma, particularly at the 1:2 dilution. For example, fentanyl at 250 ng/mL showed a CV% of 8.43%, and morphine at 100 ng/mL showed a CV% of 9.13%, indicating slightly more variation in the results. The RE% in oral fluid ranged from -14.74 to 9.58%, with some analytes showing relatively large negative deviations, such as tramadol at 200 ng/mL (RE% =−14.74%) and fentanyl at 250 ng/mL (RE% = −11.22%). These results indicate that the method effectively handles the potential challenges posed by different matrices and dilution factors. The observed deviations in some analytes are minor and do not undermine the overall performance of the method, which continues to demonstrate good accuracy and precision across both plasma and oral fluid.

**Table 8:** Dilution Integrity results in plasma and oral fluid

Analyte	Concentration (ng/mL)	Dilution factor	**Plasma** (*n *= 5)		**Oral fluid** (*n *= 5)	
			CV (%)	RE (%)	CV (%)	RE (%)
Buprenorphine	100	1:5	2.81	9.79	4.68	-7.79
	200	1:10	2.99	11.22	6.66	-9.55
	250	1:2	4.33	-4.49	9.92	-3.22
Oxycodone	100	1:5	6.00	5.11	4.81	-11.87
	200	1:10	2.85	12.51	6.04	-9.57
	250	1:2	4.53	6.18	6.73	1.63
Fentanyl	100	1:5	8.21	-5.10	3.42	1.98
	200	1:10	4.42	10.58	5.69	-6.63
	250	1:2	4.44	-11.22	8.43	-2.79
Morphine	100	1:5	7.71	-9.84	9.13	-5.40
	200	1:10	8.90	-5.21	5.98	-8.85
	250	1:2	7.45	-3.94	2.35	-12.59
Tapentadol	100	1:5	6.37	4.66	6.36	-10.00
	200	1:10	4.90	8.98	0.85	-14.74
	250	1:2	6.40	-6.79	4.72	-10.94
Tramadol	100	1:5	6.33	-10.72	9.48	2.65
	200	1:10	7.50	-6.81	9.11	-9.14
	250	1:2	6.89	-8.59	2.21	9.58

### Method applicability

The present method was successfully applied to authentic plasma and oral fluid samples obtained from patients undergoing treatment with opioids for chronic pain (paired samples). Table 9 presents several examples of the concentrations of target analytes in these samples. The analysis demonstrated the method’s ability to detect and quantify various opioids and their metabolites in plasma and oral fluid matrices.

**Table 9:** Analysis of paired authentic plasma and oral fluid samples

Sample	Analyte	Concentration (ng/mL)	
		Plasma	Oral fluid
1	Fentanyl	1.29	2.11
	Oxycodone	120.86	183.45
2	Morphine	13.44	1.98
3	Tapentadol	38.51	2.55
4	Tramadol	74.61	283.62
5	Oxycodone	20.89	4.08
	Tapentadol	360.84	58.84
6	Buprenorphine	2.76	2765.28
7	Tapentadol	32.48	154.26
	Tramadol	35.44	141.33

The number of samples to which this method was applied is too limited to establish correlations between opioid concentrations in both biological matrices. Nevertheless, the available data suggest that there are compounds for which no correlation is observed, which may have implications when if oral fluid is used as a substitute for plasma in workplace testing contexts, in accordance with SAMHSA and EWDTS guidelines [20, 21]. Therefore, it will be necessary to apply this methodology to a larger number of samples to reliably assess and establish such correlations.

**Fig. 2:**
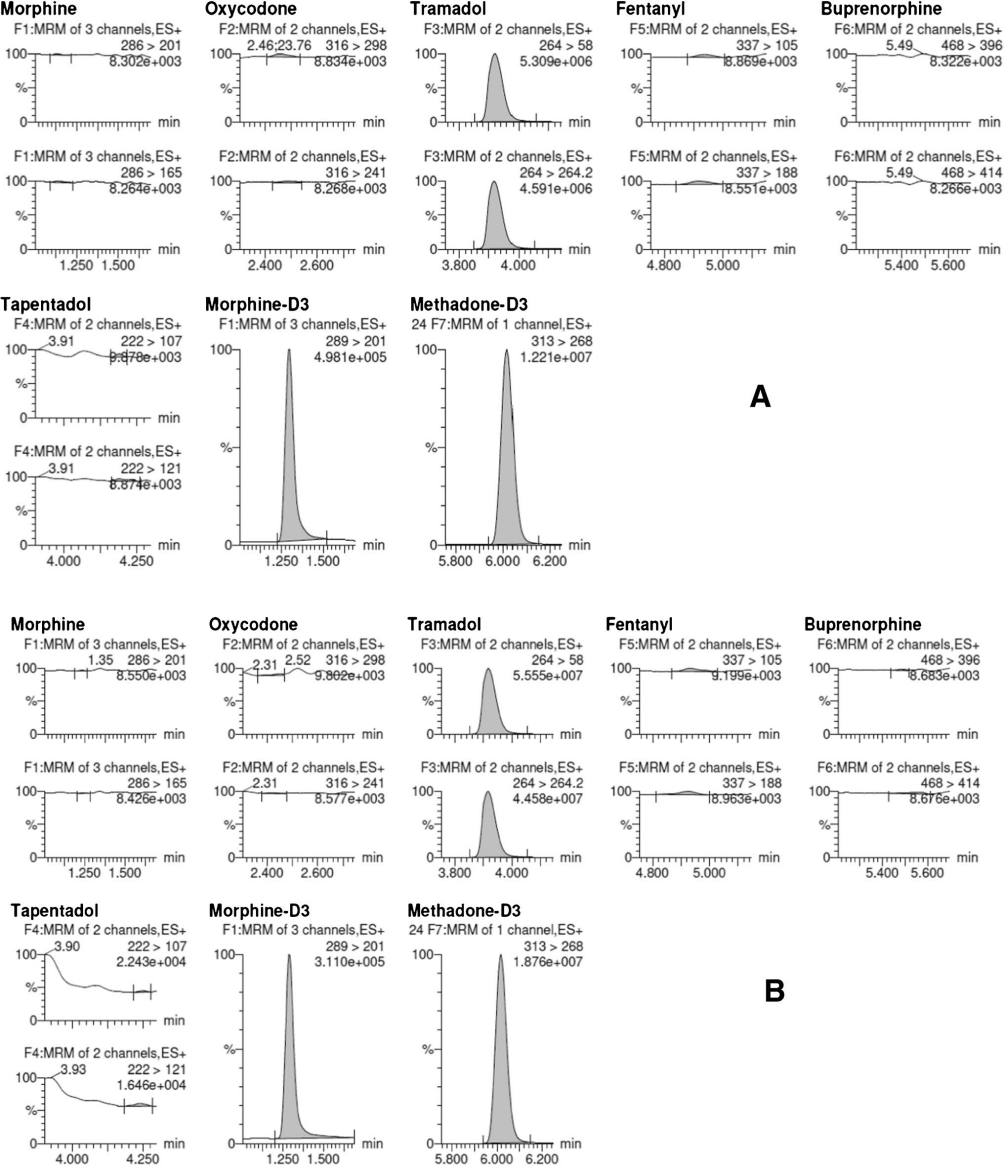
Chromatogram of sample 4 in plasma (**A**) and oral fluid (**B**)

A chromatogram of an authentic sample (sample 4) is shown in Fig. 2.

## Conclusion

In this study, a sensitive LC–MS/MS method was developed and validated for the simultaneous quantification of fentanyl, buprenorphine, tapentadol, morphine, oxycodone, and tramadol in plasma and oral fluid. The method demonstrated good linearity, precision, and accuracy, with minimal matrix effects, making it suitable for both forensic and clinical applications. It proved effective for monitoring opioids in patients undergoing chronic pain management, highlighting its potential in therapeutic drug monitoring (TDM). Oral fluid, offering a non-invasive alternative to plasma, provides insight into recent drug use, especially for ongoing opioid consumption.

The method successfully handled both low and high concentrations, including those beyond the upper limit of quantification, reinforcing its robustness. Its ability to analyse both plasma and oral fluid increases versatility, particularly for rapid and convenient drug testing. This study is the first to report the simultaneous detection of all six opioids, including tapentadol, in both matrices, filling a gap in opioid detection research. The findings support the method’s potential for improving opioid monitoring, addressing challenges in forensic toxicology, pain management, and substance use tracking, with opportunities for broader application in routine toxicological screening and TDM.

Importantly, to the best of our knowledge, no previous LC–MS/MS method has validated this complete panel of six opioids across both plasma and oral fluid using a simple protein precipitation workflow within an accredited forensic toxicology setting. The present work therefore provides a robust and routine-applicable analytical platform, complemented by preliminary observations on oral fluid–plasma behaviour of the studied compounds.

## Data Availability

All data are included in the paper.

## References

[1] Wilson N, Kariisa M, Seth P, Smith H, Davis NL. Drug and opioid-involved overdose deaths — United States, 2017–2018. MMWR Morb Mortal Wkly Rep. 2020;69:290–7. 10.15585/mmwr.mm6911a4. 32191688 10.15585/mmwr.mm6911a4 PMC7739981

[2] Polston G. Opioid overdose. In: Challenging cases and complication management in pain medicine. Cham: Springer International Publishing; 2018. pp. 3–7.

[3] Armenian P, Vo KT, Barr-Walker J, Lynch KL. Fentanyl, fentanyl analogs and novel synthetic opioids: a comprehensive review. Neuropharmacology. 2018;134:121–32. 10.1016/j.neuropharm.2017.10.016. 29042317 10.1016/j.neuropharm.2017.10.016

[4] Degenhardt L, Grebely J, Stone J, Hickman M, Vickerman P, Marshall BDL, et al. Global patterns of opioid use and dependence: harms to populations, interventions, and future action. Lancet. 2019;394:1560–79. 10.1016/S0140-6736(19)32229-9. 31657732 10.1016/S0140-6736(19)32229-9 PMC7068135

[5] Skolnick P. Treatment of overdose in the synthetic opioid era. Pharmacol Ther. 2022;233:108019. 10.1016/j.pharmthera.2021.108019. 34637841 10.1016/j.pharmthera.2021.108019

[6] Mahase E. Potent synthetic opioids are linked to rise in heroin overdoses and deaths in England. BMJ. 2023;382:1730. 10.1136/bmj.p1730. 37495235 10.1136/bmj.p1730

[7] GOV.UK. Deaths linked to potent synthetic opioids. 2025. https://www.gov.uk/government/publications/deaths-linked-to-potent-synthetic-opioids/deaths-linked-to-potent-synthetic-opioids. Accessed 4 Feb 2025.

[8] Van Amsterdam J, Pierce M, van den Brink W. Is Europe facing an emerging opioid crisis comparable to the U.S.? Ther Drug Monit. 2021;43:42–51. 10.1097/FTD.0000000000000789. 32649487 10.1097/FTD.0000000000000789

[9] Häuser W, Buchser E, Finn DP, Dom G, Fors E, Heiskanen T, et al. Is Europe also facing an opioid crisis?—A survey of European Pain Federation chapters. Eur J Pain. 2021;25:1760–9. 10.1002/ejp.1786. 33960569 10.1002/ejp.1786

[10] da Cunha KF, Rodrigues LC, Huestis MA, Costa JL. Miniaturized extraction method for analysis of synthetic opioids in urine by microextraction with packed sorbent and liquid chromatography–tandem mass spectrometry. J Chromatogr A. 2020;1624:461241. 10.1016/j.chroma.2020.461241. 32540079 10.1016/j.chroma.2020.461241

[11] Almeida E, Soares S, Gonçalves J, Rosado T, Fernández N, Rodilla JM, et al. Stability of cocaine, opiates, and metabolites in dried saliva spots. Molecules. 2022;27:641. 10.3390/molecules27030641. 35163906 10.3390/molecules27030641 PMC8839019

[12] Rosado T, Barroso M, Vieira DN, Gallardo E. Determination of selected opiates in hair samples using microextraction by packed sorbent: a new approach for sample clean-up. J Anal Toxicol. 2019;43:465–76. 10.1093/jat/bkz029. 31329881 10.1093/jat/bkz029

[13] Yasien S, Ali E, Javed M, Iqbal MM, Iqbal S, Alrbyawi H, et al. Simultaneous quantification of opioids in blood and urine by gas chromatography–mass spectrometer with modified dispersive solid-phase extraction technique. Molecules. 2022;27:6761. 10.3390/molecules27196761. 36235294 10.3390/molecules27196761 PMC9570840

[14] Cafaro A, Conti M, Pigliasco F, Barco S, Bandettini R, Cangemi G. Biological fluid microsampling for therapeutic drug monitoring: a narrative review. Biomedicines. 2023;11:1962. 10.3390/biomedicines11071962. 37509602 10.3390/biomedicines11071962 PMC10377272

[15] Gallardo E, Rosado T, Barroso M. The potential of oral fluid in drug monitoring: an update. Bioanalysis. 2023;15:657–60. 10.4155/bio-2023-0122. 37458187 10.4155/bio-2023-0122

[16] Desrosiers NA, Huestis MA. Oral fluid drug testing: analytical approaches, issues and interpretation of results. J Anal Toxicol. 2019;43:415–43. 10.1093/jat/bkz048. 31263897 10.1093/jat/bkz048

[17] Cone EJ, Huestis MA. Interpretation of oral fluid tests for drugs of abuse. Ann N Y Acad Sci. 2007;1098:51–103. 10.1196/annals.1391.043. 17332074 10.1196/annals.1384.037 PMC2700061

[18] Rosado T, Soares S, Malaca S, Gonçalves J, Barroso M, Gallardo E. The role of liquid chromatography in toxicological analysis. In: High-performance liquid chromatography: types, parameters and applications. 2018. pp. 1–120.

[19] Food and Drug Administration. Bioanalytical method validation guidance for industry. 2018.

[20] U.S. Department of Health and Human Services. Mandatory guidelines for federal workplace drug testing programs. Federal Register. 2025. https://www.federalregister.gov/documents/2025/01/16/2025-00425/mandatory-guidelines-for-federal-workplace-drug-testing-programs-authorized-testing-panels. Accessed 17 Feb 2025.

[21] Cooper G, Moore C, George C, Pichini S. Guidelines for European workplace drug testing in oral fluid. Drug Test Anal. 2011;3:269–76. 10.1002/dta.284. 21538943 10.1002/dta.284

[22] Coulter C, Garnier M, Moore C. Rapid extraction and qualitative screening of 30 drugs in oral fluid at concentrations recommended for the investigation of DUID cases. J Anal Toxicol. 2022;46:899–904. 10.1093/jat/bkac031. 35640884 10.1093/jat/bkac031

[23] Montesano C, Simeoni MC, Curini R, Sergi M, Lo Sterzo C, Compagnone D. Determination of illicit drugs and metabolites in oral fluid by microextraction on packed sorbent coupled with LC-MS/MS. Anal Bioanal Chem. 2015;407:3647–58. 10.1007/s00216-015-8583-8. 25772560 10.1007/s00216-015-8583-8

[24] Truver MT, Swortwood MJ. Quantitative analysis of novel synthetic opioids, morphine and buprenorphine in oral fluid by LC-MS/MS. J Anal Toxicol. 2018;42:554–61. 10.1093/jat/bky053. 30371839 10.1093/jat/bky053

[25] Grabenauer M, Moore KN, Bynum ND, White RM, Mitchell JM, Hayes ED, et al. Development of a quantitative LC-MS/MS assay for codeine, morphine, 6-acetylmorphine, hydrocodone, hydromorphone, oxycodone and oxymorphone in neat oral fluid. J Anal Toxicol. 2018;42:392–9. 10.1093/jat/bky021. 29554298 10.1093/jat/bky021

[26] Ares AM, Fernández P, Regenjo M, Fernández AM, Carro AM, Lorenzo RA. A fast bioanalytical method based on microextraction by packed sorbent and UPLC–MS/MS for determining new psychoactive substances in oral fluid. Talanta. 2017;174:454–61. 10.1016/j.talanta.2017.06.022. 28738608 10.1016/j.talanta.2017.06.022

[27] Bista SR, Lobb M, Haywood A, Hardy J, Tapuni A, Norris R. Development, validation and application of an HPLC-MS/MS method for the determination of fentanyl and norfentanyl in human plasma and saliva. J Chromatogr B. 2014;960:27–33. 10.1016/j.jchromb.2014.04.019. 10.1016/j.jchromb.2014.04.019 24780703

[28] Al-Qurain AA, Williams DB, Mackenzie L, Roberts MS, Wiese MD. Simultaneous LC-MS/MS quantification of oxycodone, tramadol and fentanyl and their metabolites in human plasma and whole blood collected via venepuncture and volumetric absorptive microsampling. J Pharm Biomed Anal. 2021;203:114171. 10.1016/j.jpba.2021.114171. 34087551 10.1016/j.jpba.2021.114171

[29] Goryński K, Sobczak Ł, Kołodziej D. Developing and evaluating the greenness of a reliable, all-in-one thin-film microextraction protocol for determining fentanyl, methadone, and zolpidem in plasma, urine, and oral fluid. Molecules. 2024;29:335. 10.3390/molecules29020335. 38257248 10.3390/molecules29020335 PMC10818652

[30] Musshoff F, Trafkowski J, Kuepper U, Madea B. An automated and fully validated LC-MS/MS procedure for the simultaneous determination of 11 opioids used in palliative care, with 5 of their metabolites. J Mass Spectrom. 2006;41:633–40. 10.1002/jms.1021. 16541404 10.1002/jms.1021

[31] Ito S, Mori M, Matsuo M, Yamasaki R, Oida Y, Soda M, et al. Establishment to measure oxycodone in plasma with liquid chromatography–tandem mass spectrometry. Neuropsychopharmacol Rep. 2022;42:299–305. 10.1002/npr2.12268. 35689429 10.1002/npr2.12268 PMC9515719

[32] Patel BN, Sharma N, Sanyal M, Shrivastav PS. An accurate, rapid and sensitive determination of tramadol and its active metabolite O-desmethyltramadol in human plasma by LC-MS/MS. J Pharm Biomed Anal. 2009;49:354–66. 10.1016/j.jpba.2008.10.030. 19062215 10.1016/j.jpba.2008.10.030

[33] Clavijo CF, Hoffman KL, Thomas JJ, Carvalho B, Chu LF, Drover DR, et al. A sensitive assay for the quantification of morphine and its active metabolites in human plasma and dried blood spots using high-performance liquid chromatography–tandem mass spectrometry. Anal Bioanal Chem. 2011;400:715–28. 10.1007/s00216-011-4775-z. 21400080 10.1007/s00216-011-4775-z

[34] Thomann J, Vogt SB, Guessoum A, Meyer M, Vogel M, Liechti ME, et al. Development and validation of an LC-MS/MS method for quantifying diamorphine and its major metabolites 6-monoacetylmorphine, morphine, morphine-3-glucuronide, and morphine-6-glucuronide in human plasma. J Chromatogr B. 2024;1237:124104. 10.1016/j.jchromb.2024.124104. 10.1016/j.jchromb.2024.124104 38552595

[35] Zhao W, Alshogran OY, Zhang H, Joshi A, Krans EE, Caritis S, et al. Simplified processing and rapid quantification of buprenorphine, norbuprenorphine, and their conjugated metabolites in human plasma using UPLC–MS/MS: assessment of buprenorphine exposure during opioid use disorder treatment. J Mass Spectrom. 2024;59:e5015. 10.1002/jms.5015. 38501738 10.1002/jms.5015

[36] Swortwood MJ, Scheidweiler KB, Barnes AJ, Jansson LM, Huestis MA. Simultaneous quantification of buprenorphine, naloxone and phase I and II metabolites in plasma and breastmilk by liquid chromatography–tandem mass spectrometry. J Chromatogr A. 2016;1446:70–7. 10.1016/j.chroma.2016.03.076. 27083254 10.1016/j.chroma.2016.03.076

[37] Wagner M, Bourgogne E, Varesio E, Hopfgartner G. Quantitation of polar analytes using column-switching: application to oxycodone and three metabolites in human plasma. J Chromatogr B. 2010;878:637–44. 10.1016/j.jchromb.2010.01.014. 10.1016/j.jchromb.2010.01.014 20138595

[38] Concheiro M, Gray TR, Shakleya DM, Huestis MA. High-throughput simultaneous analysis of buprenorphine, methadone, cocaine, opiates, nicotine, and metabolites in oral fluid by liquid chromatography–tandem mass spectrometry. Anal Bioanal Chem. 2010;398:915–24. 10.1007/s00216-010-3903-5. 20652688 10.1007/s00216-010-3903-5 PMC3163083

